# Nanoscale arrays of antimony telluride single crystals by selective chemical vapor deposition

**DOI:** 10.1038/srep27593

**Published:** 2016-06-10

**Authors:** Ruomeng Huang, Sophie L. Benjamin, Chitra Gurnani, Yudong Wang, Andrew L. Hector, William Levason, Gillian Reid, C. H. (Kees) De Groot

**Affiliations:** 1 Electronics and Computer Science, University of Southampton, SO17 1BJ UK; 2 Chemistry, University of Southampton, SO17 1BJ UK; 3 School of Natural Sciences, Mahindra École Centrale, India

## Abstract

Arrays of individual single nanocrystals of Sb_2_Te_3_ have been formed using selective chemical vapor deposition (CVD) from a single source precursor. Crystals are self-assembled reproducibly in confined spaces of 100 nm diameter with pitch down to 500 nm. The distribution of crystallite sizes across the arrays is very narrow (standard deviation of 15%) and is affected by both the hole diameter and the array pitch. The preferred growth of the crystals in the <1 1 0> orientation along the diagonal of the square holes strongly indicates that the diffusion of adatoms results in a near thermodynamic equilibrium growth mechanism of the nuclei. A clear relationship between electrical resistivity and selectivity is established across a range of metal selenides and tellurides, showing that conductive materials result in more selective growth and suggesting that electron donation is of critical importance for selective deposition.

Unique layered geometries and remarkable mechanical and electronic properties are some of the features that make chalcogenide materials highly promising candidates in a variety of applications^1^,^2^,^3^,^4^,^5^. Sb_2_Te_3_ is one of the most important binary chalcogenides due to its narrow band-gap and the anisotropy of its material properties. It has been the subject of great interest over recent years owing to its potential applications as a thermoelectric material, in phase change memory and as a topological insulator^6^,^7^,^8^. For these applications, better performances are observed when scaling the chalcogenide material down to the nanoscale. Nanostructured chalcogenides (including Sb_2_Te_3_) have shown significantly enhanced thermoelectric efficiencies due to quantum confinement effects and reductions in lattice thermal conductivity^9^. To fully realize their technological potentials, the metal chalcogenides have to be fabricated into complex nanostructures within the electronic and optoelectronic devices^10^. This normally uses traditional microfabrication techniques involving lithography and etching processes which could induce damage to the material, and reduce the performance of the devices^2^,^11^. The controlled production of high-quality nanoscale chalcogenides in precise locations is therefore an essential, although highly challenging, task. Tuneable selective growth of these materials, especially on the nanoscale, is therefore highly desirable.

Chemical vapor deposition (CVD) enables the production of thin films with superior quality compared to those obtained by sputtering in terms of conformity, coverage and stoichiometry control^12^. Many metal chalcogenide materials can be deposited *via* CVD techniques and this has stimulated a large amount of work on the development of tailored CVD precursors for their production^13^,^14^,^15^,^16^,^17^. CVD has mostly been used to achieve planar films rather than selective hole filling, and area selective growth is not a natural feature of CVD. Nevertheless, selective deposition onto one surface in the presence of another surface is still possible. The selective CVD of metals has been comprehensively reviewed by Gladfelter and Hampden-Smith *et al.* and a variety of mechanisms proposed^18^,^19^,^20^. The selective epitaxial growth of SiGe in the source/drain area for Si transistors is also well established^21^,^22^. A number of reports are also found on the selective CVD of chalcogenide materials. Choi *et al.* reported the selective growth of stoichiometric GeSbTe on the TiN plug material in a contact hole formed in a SiO_2_ dielectric layer by using multiple precursors^23^. Eom *et al.* demonstrated the selective deposition of (GeTe_2_)_(1−x)_(Sb_2_Te_3_)_x_ into 120 nm diameter pores by atomic layer deposition^24^. Position-controlled synthesis of single-crystal chalcogenides nanoplate arrays on mica substrates has also been reported using van der Waals epitaxy^25^,^26^. We recently showed the selective CVD of a range of binary chalcogenides, including Sb_2_Te_3_, using single source precursors^27^,^28^,^29^,^30^,^31^. The ability to deposit alloys using single source reagents can be advantageous as it can offer improved control of stoichiometry and fewer defects, while the single source precursors themselves are often less toxic and easier to handle compared to those used in dual source CVD. However, the implementation of selective deposition in a wider range of applications is hindered by the limited knowledge on the origins of selectivity. A better understanding of the selectivity mechanism will therefore be beneficial for achieving better selective deposition performances over a wider range of materials.

Here we report the deposition of individual Sb_2_Te_3_ nanocrystals from a one-step low pressure (LP) CVD method using a carefully designed single source precursor. This approach allows selective deposition of single Sb_2_Te_3_ nanocrystals into nanoscale arrays with different pitches, geometries and hole diameters. This selectivity is shown to prevail over different surface combinations. The effect of both array pitch and hole diameter on the crystal size will be studied. Furthermore, the origins for this selectivity are investigated by comparison with our previous work on selective deposition of several binary chalcogenides. The possible mechanisms with respect to both the substrate and material properties will be discussed.

## Results

**Figure 1:**
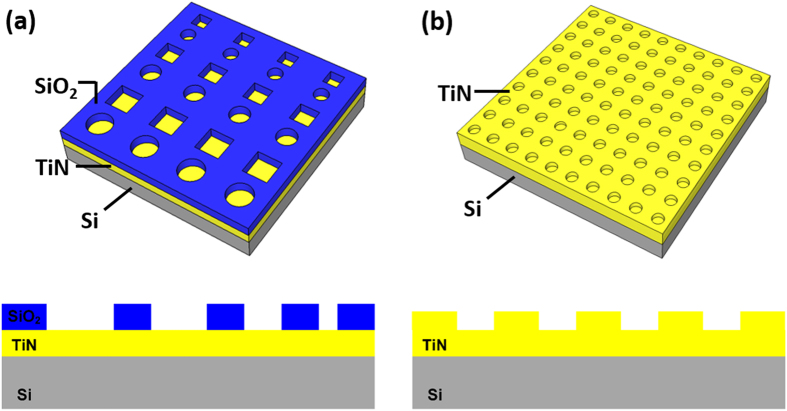
Illustrations and the cross-section views of the lithographically patterned (**a**) SiO_2_/TiN substrate and (**b**) 1etch-TiN/2etch-TiN substrate.

Patterned substrates with different material combination (SiO_2_/TiN) and different surface preparation combination (one-step etch TiN/ two-step etch TiN) were fabricated in order to investigate the different selective deposition behavior. Illustrations of the patterned substrate, as well as a cross-section view, are shown in Fig. 1.

**Figure 2:**
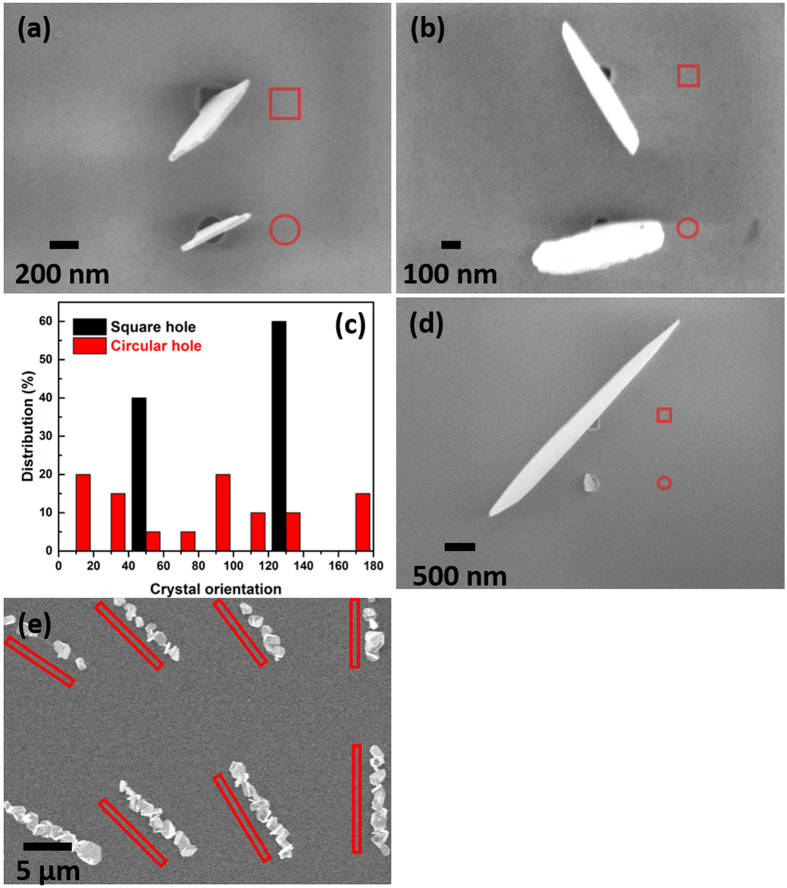
Individual nano-crystals of Sb_2_Te_3_ contacting through a crystal edge to the TiN surface within 200 nm (**a**) and 100 nm (**b**) diameter holes on the substrate; (**c**) Distribution of crystal orientations in both square and circular holes; (**d**) example of crystal growth in 200 nm square hole (top) and 200 diameter circular hole (bottom); (**e**) Sb_2_Te_3_ single crystals grown within 10 μm long, random oriented trenches with width of 100 nm (top) and 200 nm (down). Contours of the holes and trenches are placed by the side to facilitate reading.

The deposition of Sb_2_Te_3_ onto SiO_2_/TiN patterned substrates resulted in high selectivity for deposition onto the TiN surface inside the hole structure. Figure 2a,b show the Sb_2_Te_3_ single nano-crystals selectively deposited into 200 nm and 100 nm TiN holes (both square and circular), with no deposition observed on the surrounding SiO_2_ surface. It is worth mentioning that this preferred deposition onto the conductive surface is particularly favoured in the applications such as memory and thermoelectric devices^1^,^2^. Within the square holes (upper hole in Fig. 2a,b), the nano-crystal will grow by aligning itself to the diagonal direction of the square to achieve the largest size, whereas in the case of circular holes (lower hole in Fig. 2a,b), the in-plane orientation of the nano-crystals is random. A statistic distribution of the crystal orientation is presented in Fig. 2c by measuring the angles of the crystal orientation in these two types of holes. While crystals in square holes show a distribution only in two diagonal directions (*ca.* 45° and 135°), the distribution of crystals in circular holes are random. This suggests that the crystal orientation is affected by the geometry of the hole rather than the substrate material as the TiN layer is not epitaxially aligned to the Si substrate. The existence of this artificial epitaxial configuration within the square holes results from the interplay between thermodynamic (diffusion) and kinetic effects^32^ in which the former dominates in this case. The adatoms on the hole terrace are free to diffuse due to the relatively low energy barrier and align themselves to achieve the maximum crystal size in the <1 1 0> preferred orientation. Furthermore, it is possible that the diffusion of the adatoms within the corners is less favoured due to the relatively high energy barrier^33^, or that corners could act as preferential nucleation sites due to their increased coordination compared to the terrace sites^34^,^35^. The diffusion on the terrace stops when it hits the critical nuclei at the corners and condenses there, leading to the diagonal growth of the crystals within the square hole. Figure 2c shows the growth of the crystal in diagonal direction within the square hole is usually considerably larger than that in a circular hole due to free diffusion of the adatoms. Figure 2d shows Sb_2_Te_3_ crystals grown within 10 μm long trenches of random orientations. The lack of orientation growth and the limited size of the crystals within the trenches suggest that that corner nucleation might play a role or that the effect of diffusion is diminishing with increasing dimension.

**Figure 3:**
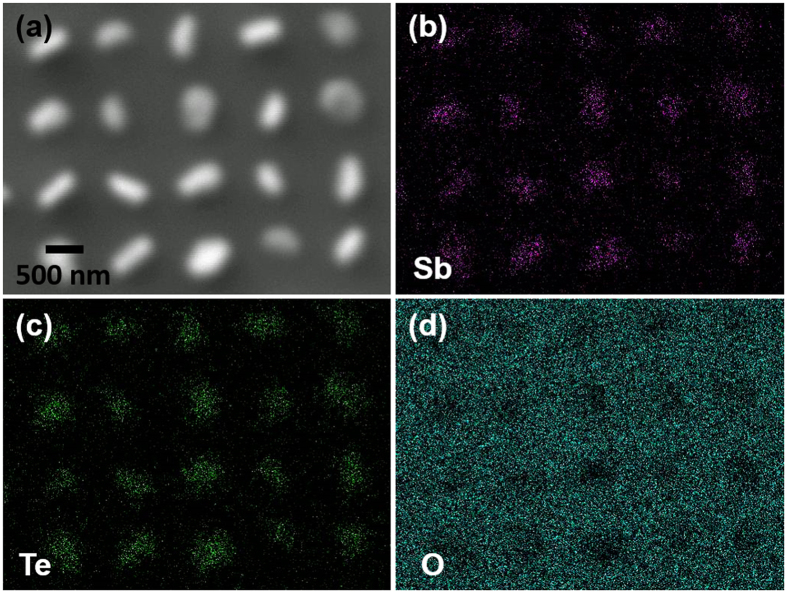
SEM image (**a**) and EDX element maps (**b**–**d**) confirming the selective deposition of Sb_2_Te_3_ occurring only within the holes (200 nm) with growth preferentially onto the TiN surface.

EDX mapping was applied to the array of Sb_2_Te_3_ single nano-crystals deposited on this SiO_2_/TiN substrate with circular hole arrays as shown in Fig. 3. It is clearly demonstrated that both Sb and Te have been deposited into the TiN holes (200 nm). No deposition is found on the surrounding SiO_2_ surfaces.

**Figure 4:**
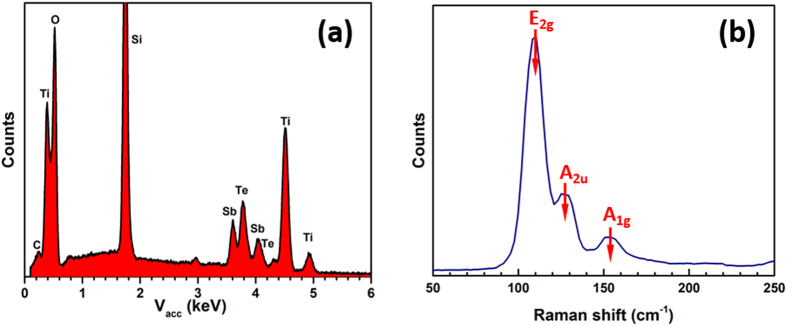
(**a**) EDX spectrum of a Sb_2_Te_3_ single nano-crystal in the hole; (**b**) Raman spectrum of the Sb_2_Te_3_ dense array.

The composition of these Sb_2_Te_3_ single nano-crystals was confirmed by quantitative EDX analysis. Figure 4a is an EDX spectrum obtained from a single nano-crystal, showing both Sb and Te in the correct ratio with no obvious impurities, except for a trace of carbon. The array of Sb_2_Te_3_ was also characterised by Raman spectroscopy, the result revealing three main peaks positioned at 120, 138 and 165 cm^−1^, as shown in Fig. 4b. These match well with the Raman spectrum obtained from thin films deposited by the same method^31^, and are in agreement with the reported E_2g_, A_2u_ and A_1g_ vibration modes of Sb_2_Te_3_^36^.

**Figure 5:**
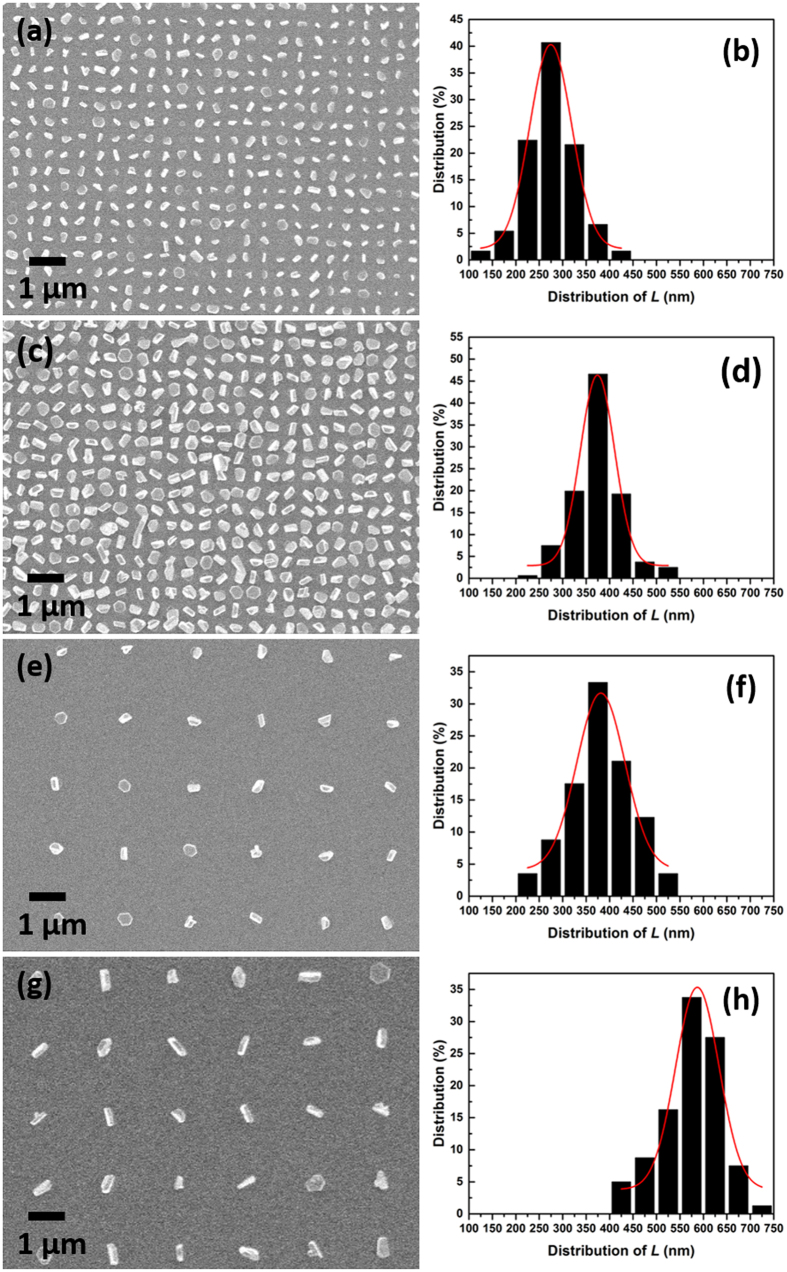
SEM image (**a**,**c**,**e**,**g**) and the corresponding distribution of the planar projection of longest axis (*L*) (**b**,**d**,**f**,**h**) of Sb_2_Te_3_ single nano-crystal arrays grown selectively onto the etched TiN/TiN substrates with circular holes by CVD. The hole diameters are 100 nm (**a**,**e**) and 200 nm (**c**,**g**), respectively. The array pitches are 500 nm (**a**,**c**) and 2000 nm (**e**,**g**), respectively.

In addition to the selectivity observed on the SiO_2_/TiN substrates, deposition onto the 1etch-TiN/2etch-TiN patterned substrates resulted in unprecedentedly high selectivity, as shown in Fig. 5. Here four arrays with different circular hole diameters and pitches were patterned on the same substrate. It can be clearly observed that within all of the arrays, every hole is occupied by one Sb_2_Te_3_ nano-crystal, with no deposit on the surrounding surface. The planar projection of the longest axis (*L*) of each crystal on the SEM image is measured and used in this work for the description of the crystal length and distribution as summarised in Table 1. The effect of hole diameter on the crystal length is investigated by comparing the crystal size between Fig. 5a,c, where the array pitch is the same (500 nm), but the hole diameters are 100 nm and 200 nm, respectively. It can be observed that crystals are typically larger in larger holes compared with those in smaller holes. This corresponds well the crystal length distributions in Fig. 5b,d. Similar behavior can also be observed when comparing crystals in Fig. 5e,g. This result is not unexpected considering that CVD reaction in the holes with smaller diameter is limited by the surface area, resulting in smaller crystals.

The crystal size is also affected by the array pitch, with a larger pitch leading to larger crystal size. This can be proved by comparing Fig. 5a,b with Fig. 5e,f, where the *L* increase substantially with the increase of pitch from 500 nm to 2000 nm, even though the hole diameter remains constant (100 nm). This is also observed for 200 nm holes with different pitches (Fig. 5c,d and Fig. 5g,h). This effect of the array pitch can be explained by the mass transport of precursor for each hole. Although the precursor flow is similar over those adjacent arrays, the actual precursor density for each hole is determined by the number of holes within each array. A smaller number (larger pitch) will lead to larger mass transport for each hole, and hence larger crystals. Consistent with this, for the arrays in Fig. 2, where the pitch is much larger (10000 nm), the crystals are very much larger than all those in Fig. 5.

Narrow distributions of crystal sizes can be observed in all images. The standard deviation of the average length *L* for deposited Sb_2_Te_3_ nano-crystals in these restricted arrays is *ca.* 15%. For sparse arrays, a much larger standard deviation is obtained (*ca.* 40%).

## Discussion

The investigation of the potential mechanism for this selective deposition behavior considers both the contribution from the substrate properties and the material properties. The results from our previous work on the selective deposition of binary chalcogenides are incorporated in the discussion.

### Substrate selectivity

**Figure 6:**
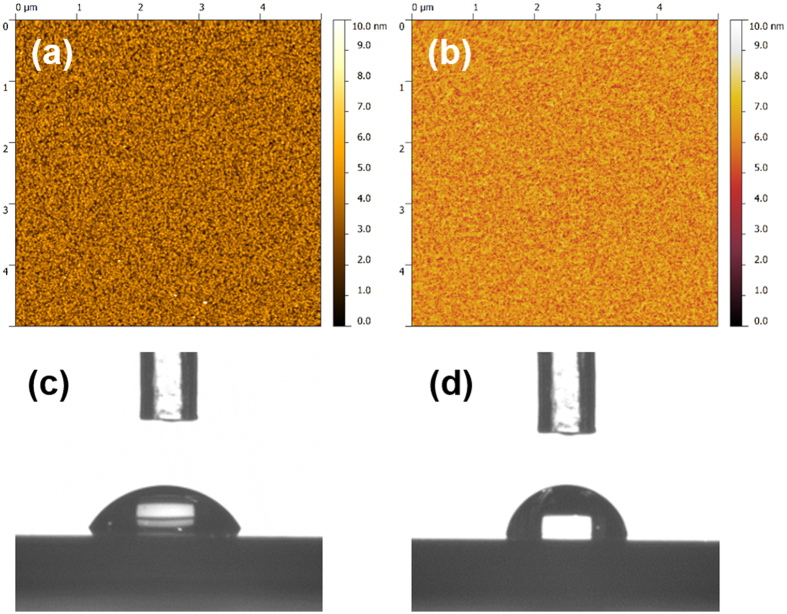
AFM measurements on the SiO_2_ (**a**) and TiN (**b**) surfaces within the SiO_2_/TiN substrate. The resulting average roughnesses are 0.71 nm and 0.60 nm, respectively; Contact angle measurements on the SiO_2_ (**c**) and TiN (**d**) surface. The resulting angles are 57.6° and 72.2°, respectively.

Substrate properties play an important role in the selective deposition behavior. In the case of SiO_2_/TiN patterned substrates, the deposition of Sb_2_Te_3_ was preferred onto the TiN surface rather than the SiO_2_ surface. Similar selectivity was also observed in our previous work for Ga_2_Te_3_, SnSe_2_, TiSe_2_ and Bi_2_Te_3_^27^,^28^,^29^,^30^. We have previously excluded the effect of the hole geometry on the selectivity as large areas up to mm size were still filled selectively. Same experiments were also attempted on SiO_2_/SiO_2_ patterned substrates where no selectivity was observed. Surface roughness can also be excluded from being responsible for this selectivity. AFM measurements on the SiO_2_ and TiN surfaces within this SiO_2_/TiN substrate reveal both are very smooth films with the average roughness R_a_ being 0.71 nm and 0.60 nm, respectively, as shown in Fig. 6a,b. The surface hydrophilicity of these surfaces was studied by contact angle measurements. Patterned substrates cannot be directly used for this experiment as the hole sizes are too small for the water droplets. Instead, thin film substrates were used, with the TiN surface being treated briefly in the RIE process to represent their real surface conditions in the patterned substrates. The measured contact angles for the SiO_2_ and TiN surfaces were 57.6° and 72.2°, respectively, as shown in Fig. 6c,d. This indicates a correlation with the selectivity observed, suggesting that the deposition has a tendency to happen on the surface which is more hydrophobic, as we have noted previously^28^. A possible explanation is that this hydrophobic TiN surface facilitates the adsorption due to presence of the hydrophobic butyl groups in the precursor molecules. Similar selective behavior over hydrophobic surfaces was also reported in the chemical vapor deposition of Al and Pt^37^.

Preferred deposition onto TiN over SiO_2_ was also reported by Choi *et al.* where the substrate dependent growth behavior was attributed to the electron donation from the substrate. They showed that surfaces with more free electrons have a much higher growth rate for GeSbTe, compared to insulating surfaces^38^. It is suggested that a more conductive surface could help to enhance precursor decomposition and the removal of ligands from the adsorbed precursor molecules. This facilitates the formation of surface complexes which work as the nuclei for the growth of crystals. Although it is widely acknowledged that a mixed TiO_x_N_y_ composition will normally form on the surface of the TiN, due to exposure to air^39^, the conductivity of such a surface should still be much higher than that of the SiO_2_ surface, providing enough discrimination for the selective deposition behavior. In addition, when the metal oxides (e.g. TiO_x_ in this case) are reduced by the precursors, it is reported that either they or their reduced products can catalyse the further growth of the nuclei^40^.

**Figure 7:**
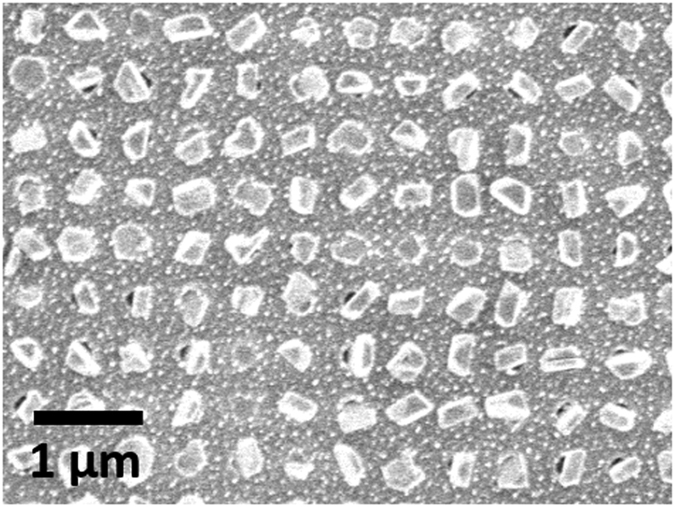
SEM image of Sb_2_Te_3_ single nano-crystal array grown by CVD onto patterned SiO_2_/TiN substrates with circular nanoscale holes under excess precursor flow.

However, it is worth mentioning that the mechanisms proposed above operate within a certain temperature range and precursor flow density window. An excess of precursor flow density does result in the formation of nuclei on the SiO_2_ surface as demonstrated in Fig. 7. The formation of this high density of stable nuclei on both surfaces implies that nucleation is fast and that the surface diffusion of adatoms is smaller on the SiO_2_ than on the TiN^41^. The growth of larger aggregates on the TiN surface resulting in crystals within the TiN holes that are substantially larger than the nuclei on the SiO_2_ surface. This too suggests the growth of the Sb_2_Te_3_ crystals is a thermodynamic (diffusion) dominated process, which corresponds well with the conclusions based on the orientation of the crystals as shown in Fig. 2. This difference of surface diffusivities between different surfaces has also reported for InAs on AlAs/GaAs facets^42^.

The selective deposition behavior on the 1etch-TiN/2etch-TiN patterned substrates was also investigated. TiN surfaces under different RIE treatment times were studied using both AFM and hydrophilicity measurements. No significant differences between the etched and unetched surfaces were observed in either measurement, suggesting that the surface hydrophilicity and roughness are not responsible for the selectivity observed using this substrate. One possible explanation could be that the TiN surface within the hole has been more heavily treated under plasma, resulting in a more defective TiO_x_N_y_ surface with more free electrons.

### Material selectivity

The role of materials’ electrical properties in this selective deposition behavior was studied by reviewing the results of six different binary chalcogenide materials deposited on the same types of substrates. Only the selectivity on the SiO_2_/TiN patterned substrate is considered. The electrical properties of these six chalcogenides were reported in our previous work, based on thin film samples, and are listed in Table 2. A wide range of material resistivities can be observed, from 10^−4^ Ω∙cm to 10^3^ Ω∙cm, which corresponds well with the material band-gaps reported^43^.

**Figure 8:**
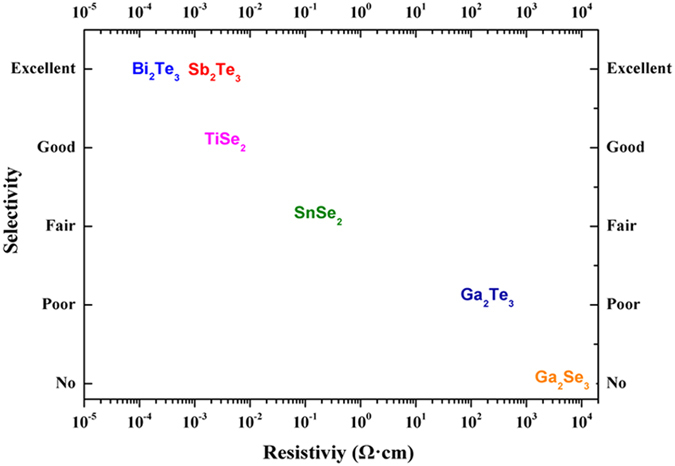
Selectivity of single source precursor chemical vapor deposited binary chalcogenides as a function of material resistivity with respect to the growth on the SiO_2_/TiN substrate. See text for the explanation of the y-axis.

The selective performance onto the SiO_2_/TiN patterned substrate is evaluated by comparing the smallest TiN hole size for which selectivity is observed, together with the temperature and precursor flow window. No selectivity was observed for Ga_2_Se_3_, with both the TiN and SiO_2_ surfaces covered after deposition. LPCVD of Ga_2_Te_3_ showed a preference for deposition onto the TiN over the SiO_2_ surface. However, the selectivity can only be observed on large TiN surfaces (>50 μm) in a very narrow temperature and precursor flow window. The selective deposition behavior is much more prominent for SnSe_2_ and TiSe_2_ where the smallest hole size diameters for which selective deposition was observed are 5 μm and 2 μm, respectively. The most highly selective performance was achieved for both Bi_2_Te_3_ and Sb_2_Te_3_, where the selectivity was still clearly evident in the nanometre region. The scales on which selectivity is found are plotted against the resistivity of each material in Fig. 8. It is clear that a low resistivity is correlated with a higher deposition selectivity. The electron donation mechanism serves well in explaining this trend. Although the deposition rate of the first few layers might be directly linked to the surface properties, to establish a preferred deposition onto the TiN surface the subsequent growth must be determined by the material’s own properties. A more resistive material would have less free electrons on the surface and its growth rate would become self-limited, leading to a less selective behavior. As selective growth requires both substrate selectivity and self-selectivity, this indicates that this electron donation process is of paramount importance.

## Conclusion

Highly selective deposition of dense arrays of Sb_2_Te_3_ single nano-crystals has been demonstrated onto both SiO_2_/TiN and etched TiN/TiN nanoscale-patterned substrates. Crystal growth is preferred in confined holes of 100 nm diameter with pitch down to 500 nm. The crystal size is dependent on the hole size as well as the array pitch. The distribution of the crystal size across the array is very narrow. The preferred growth of the crystals at <1 1 0> orientation along the diagonal of the square holes strongly indicates that the diffusion of adatoms, rather than the deposition, is the predominant growth mechanism of the nuclei.

Selective deposition of a range of binary chalcogenides are all found to be preferred onto the TiN surface of the SiO_2_/TiN patterned substrates. The level of selectivity is affected by the material resistivity, with a less resistive material resulting in a higher level of selectivity. The potential mechanisms for this selective deposition behavior have been investigated. Surface hydrophilicity as well as conductivity are suggested to be the key factors. The effect of electron donations is believed to play a vital part in both the substrate selectivity and material selectivity.

## Methods

### Precursor preparation and characterization

The single source precursor MeSb(Te^n^Bu)_2_ (Me = CH_3_, ^n^Bu = n-C_4_H_9_) was synthesized and characterized as reported previously^31^.

### Substrate preparation

Patterned substrates with different material combination (SiO_2_/TiN) and different surface preparation combination (one-step etch TiN/ two-step etch TiN) were fabricated in order to investigate the different selective deposition behavior. Illustrations of the patterned substrate, as well as a cross-section view, are shown in Fig. 1. For the patterned SiO_2_/TiN substrate, a TiN thin film was first deposited on a silicon substrate by using medium frequency plasma assisted magnetron sputtering (Leybold Optics HELIOS Pro XL) at room temperature. In this process, the substrate was rotating at a speed of 180 rpm to ensure a uniform deposition. During each rotation, a thin layer of Ti was firstly deposited from dual magnetron metal targets (99.99% purity) using a power of 3000W in an Ar atmosphere. This thin film was converted to titanium nitride by passing the substrate under an N_2_ plasma produced by the RF source. The insulating SiO_2_ film was subsequently sputtered on top of the TiN layer using the same technique with pure Si targets (99.99% purity). This SiO_2_ layer was then patterned by e-beam lithography. Reactive ion etching (RIE) was used to etch the exposed SiO_2_ surface to create the hole structure, with diameters ranging from 100 nm to 500 nm. This was performed by a RIE80+ with CHF_3_ and Ar. One extra minute of over-etch was normally applied to ensure the complete removal of SiO_2_ from the different feature sizes. The TiN layer underneath served as an etch stop. In the case of the 1etch-TiN/2etch-TiN patterned substrates, a similar fabrication process was adopted without the SiO_2_ layer, in order to separate geometric and processing effects from the effects of the material choice. RIE with CHF_3_ and Ar were used for the etching, and each etch step lasted for 1 minute.

### Substrate Characterisation

Properties of the substrates were measured to investigate the underlying mechanism of the selective deposition behavior. Surface roughness was measured by atomic force microscopy (AFM) using a Veeco Dimension 3100 in tapping mode. Contact angles of different surfaces were determined using a Krauss DSA100 Drop Shape Analyser in a purpose-built arrangement assembled on a vibrationally isolated platform. The drop of water was expelled through a microsyringe onto the surface of the substrate. The contact angle *θ* was measured using a microscope equipped with a goniometer. The contact angle was estimated as the tangent normal to the drop at the intersection between the sessile drop and the surface.

### LPCVD onto patterned substrates

For a typical deposition, both precursor and substrates were loaded into a closed-end quartz tube in a glove-box. The precursor, normally *ca.* 5 mg, was placed at the closed end and several patterned substrates were positioned end-to-end near the precursor. After loading the substrates, the tube was set to 450 °C in a furnace such that the precursor was outside the heated zone; the tube was evacuated, heated to the set temperature under 0.05 mmHg and the furnace was allowed to stabilise. The tube position was subsequently adjusted so that the precursor was moved gradually towards the hot zone until evaporation was observed. The tube remained unmoved until the precursor had completely evaporated (no residual precursor remained), i.e. *ca.* 1–3 h. After this, the tube was cooled to room temperature and transferred to the glove box where the tiles were removed and stored under an N_2_ atmosphere prior to analysis.

### Characterisation of the Sb_2_Te_3_ nanocrystals

The deposited Sb_2_Te_3_ nanocrystals were investigated using scanning electron microscopy (SEM) and energy dispersive X-ray (EDX) spectroscopy. A Zeiss EVO LS 25 microscope equipped with an Oxford INCA x-act X-ray detector was used for the SEM and EDX analyses. High resolution SEM measurements were carried out with a field emission SEM (Jeol JSM 7500F).

## Additional Information

**How to cite this article**: Huang, R. *et al.* Nanoscale arrays of antimony telluride single crystals by selective chemical vapor deposition. *Sci. Rep.* **6**, 27593; doi: 10.1038/srep27593 (2016).

## Figures and Tables

**Table 1:** Average length *L* of deposited Sb_2_Te_3_ nano-crystals in arrays with different hole diameters and pitches.

Pitch (nm)	Hole diameter (nm)	Average length *L*(nm)
500	100	276 ± 53
500	200	373 ± 49
2000	100	379 ± 63
2000	200	575 ± 63
10000	100	1000 ± 400
10000	200	1000 ± 400

**Table 2:** Singe source precursor used for LPCVD of the six binary chalcogenide materials and the electrical properties obtained from the deposited thin films in previous work.

Materials	Precursors (Me = CH_3_, ^n^Bu = n-C_4_H_9_)	Resistivity (Ω∙cm)	Carrier density (cm^−3^)	Mobility (cm^2^/V∙s)	Band-gap (eV) ^43^
Ga_2_Se_3_^27^	GaCl_3_(Se^n^Bu_2_)	(9.0 ± 1.0) × 10^3^	2 × 10^13^	20–80	2.0
Ga_2_Te_3_^27^	GaCl_3_(Te^n^Bu_2_)	(2.0 ± 0.1) × 10^2^	5 × 10^15^	10–40	1.1
SnSe_2_^28^	SnCl_4_{^n^BuSe(CH_2_)_2_Se^n^Bu}	(2.1 ± 0.1) × 10^−1^	5 × 10^18^	2–6	0.97
TiSe_2_^29^	TiCl_4_(^n^Bu_2_Se)_2_	(3.4 ± 0.1) × 10^−3^	1 × 10^22^	0.2	0.15
Bi_2_Te_3_^30^	BiCl_3_(Te^n^Bu_2_)_3_	(5.6 ± 0.1) × 10^−4^	2 × 10^20^	56.6	0.16
Sb_2_Te_3_^31^	MeSb(Te^n^Bu)_2_	(9.9 ± 0.8) × 10^−4^	5 × 10^19^	138	0.28

## References

[b1] Gao M.-R., Xu Y.-F., Jiang J. & Yu S.-H. Nanostructured metal chalcogenides: synthesis, modification, and applications in energy conversion and storage devices. Chem. Soc. Rev. 42, 2986–3017 (2013). 23296312 10.1039/c2cs35310e

[b2] Hudgens S. & Johnson B. Overview of phase-change chalcogenide nonvolatile memory technology. MRS Bull. 29, 829–832 (2004).

[b3] Hasan M. Z. & Kane C. L. Colloquium: Topological insulators. Rev. Mod. Phys. 82, 3045–3067 (2010).

[b4] Maignan A., Guilmeau E., Gascoin F., Bréard Y. & Hardy V. Revisiting some chalcogenides for thermoelectricity. Sci. Technol. Adv. Mater. 13, 053003 (2012). 10.1088/1468-6996/13/5/053003 PMC5099614 27877513

[b5] Eggleton B. J., Luther-Davies B. & Richardson K. Chalcogenide photonics. Nat. Photonics 5, 141–148 (2011).

[b6] Goncalves L. M., Alpuim P., Rolo A. G. & Correia J. H. Thermal co-evaporation of Sb_2_Te_3_ thin-films optimized for thermoelectric applications. Thin Solid Films 519, 4152–4157 (2011).

[b7] Zhu M. *et al.* Uniform Ti-doped Sb_2_Te_3_ materials for high-speed phase change memory applications. Appl. Phys. Lett. 104, 1–6 (2014).

[b8] Zhu J. *et al.* Superconductivity in topological insulator Sb_2_Te_3_ induced by pressure. Sci. Rep. 3, 2016 (2013). 23783511 10.1038/srep02016 PMC3687246

[b9] Vineis C. J., Shakouri A., Majumdar A. & Kanatzidis M. G. Nanostructured thermoelectrics: Big efficiency gains from small features. Adv. Mater. 22, 3970–3980 (2010). 20661949 10.1002/adma.201000839

[b10] Wang Q. H., Kalantar-Zadeh K., Kis A., Coleman J. N. & Strano M. S. Electronics and optoelectronics of two-dimensional transition metal dichalcogenides. Nat. Nanotechnol. 7, 699–712 (2012). 23132225 10.1038/nnano.2012.193

[b11] Fonash S. J. An overview of dry etching damage and contamination effects. J. Electrochem. Soc. 137, 3885–3892 (1990).

[b12] Choy K. L. Chemical vapour deposition of coatings. Prog. Mater. Sci. 48, 57–170 (2003).

[b13] Ahn J.-K. *et al.* Metalorganic chemical vapor deposition of non-GST chalcogenide materials for phase change memory applications. J. Mater. Chem. 20, 1751–1754 (2010).

[b14] Waters J., Crouch D., Raftery J. & O’Brien P. Deposition of bismuth chalcogenide thin films using novel single-source precursors by metal-organic chemical vapor deposition. Chem. Mater. 16, 3289–3298 (2004).

[b15] Huang C. C., Gholipour B., Ou J. Y., Knight K. & Hewak D. W. Electrical phase change of CVD-grown Ge-Sb-Te thin-film device. Electron. Lett. 47, 288–289 (2011).

[b16] Abrutis A. *et al.* Chemical vapor deposition of chalcogenide materials for phase-change memories. Microelectron. Eng. 85, 2338–2341 (2008).

[b17] Abrutis A. *et al.* Hot-Wire Chemical Vapor Deposition of Chalcogenide Materials for Phase Change Memory Applications. Chem. Mater. 20, 3557–3559 (2008).

[b18] Gladfelter W. Selective metalization by chemical vapor deposition. Chem. Mater. 5, 1372–1388 (1993).

[b19] Hampden‐Smith M. & Kodas T. Chemical vapor deposition of metals: Part 1. An overview of CVD processes. Chem. Vap. Depos. 1, 8–23 (1995).

[b20] Hampden-Smith M. J. & Kodas T. T. Chemical vapor deposition of metals: Part 2. Overview of selective CVD of Metals. Chem. Vap. Depos. 1, 39–48 (1995).

[b21] Vescan L. Selective epitaxial growth of SiGe alloys—influence of growth parameters on film properties. Mater. Sci. Eng. B 28, 1–8 (1994).

[b22] Ning X. J. *et al.* Selective epitaxial growth of SiGe for strained Si transistors. Mater. Sci. Eng. B Solid-State Mater. Adv. Technol. 134, 165–171 (2006).

[b23] Choi B. J. *et al.* Combined atomic layer and chemical vapor deposition, and selective growth of Ge_2_Sb_2_Te_5_ films on TiN/W contact plug. Chem. Mater. 19, 4387–4389 (2007).

[b24] Eom T. *et al.* Conformal formation of (GeTe_2_)_(1– x)_(Sb_2_Te_3_)_x_ layers by atomic layer deposition for nanoscale phase change memories. Chem. Mater. 24, 2099–2110 (2012).

[b25] Li H. *et al.* Controlled synthesis of topological insulator nanoplate arrays on mica. J. Am. Chem. Soc. 134, 6132–5 (2012). 22455625 10.1021/ja3021395

[b26] Lin M. *et al.* Controlled growth of atomically thin In_2_Se_3_ flakes by van der Waals epitaxy. J. Am. Chem. Soc. 135, 13274–13277 (2013). 23978251 10.1021/ja406351u

[b27] George K. *et al.* Telluroether and selenoether complexes as single source reagents for low pressure chemical vapor deposition of crystalline Ga_2_Te_3_ and Ga_2_Se_3_ thin films. Chem. Mater. 25, 1829–1836 (2013).

[b28] de Groot C. H. *et al.* Highly selective chemical vapor deposition of tin diselenide thin films onto patterned substrates via single source diselenoether precursors. Chem. Mater. 24, 4442–4449 (2012).

[b29] Benjamin S. L. *et al.* Area selective growth of titanium diselenide thin films into micropatterned substrates by low-pressure chemical vapor deposition. Chem. Mater. 25, 4719–4724 (2013). 24489437 10.1021/cm402422e PMC3903341

[b30] Benjamin S. L. *et al.* Controlling the nanostructure of bismuth telluride by selective chemical vapour deposition from a single source precursor. J. Mater. Chem. A 2, 4865–4869 (2014).

[b31] Benjamin S. L. *et al.* Chemical vapour deposition of antimony chalcogenides with positional and orientational control: precursor design and substrate selectivity. J. Mater. Chem. C 3, 423–430 (2015).

[b32] Barth J. V., Costantini G. & Kern K. Engineering atomic and molecular nanostructures at surfaces. Nature 437, 671–679 (2005). 16193042 10.1038/nature04166

[b33] Briseno A. L. *et al.* Patterning organic single-crystal transistor arrays. Nature 444, 913–917 (2006). 17167482 10.1038/nature05427

[b34] Gambardella P., Blanc M., Brune H., Kuhnke K. & Kern K. One-dimensional metal chains on Pt vicinal surfaces. Phys. Rev. B 61, 2254–2262 (2000).

[b35] Wang W. & Chi L. Area-selective growth of functional molecular architectures. Acc. Chem. Res. 45, 1646–1656 (2012). 22830409 10.1021/ar200299w

[b36] Sosso G. C., Caravati S. & Bernasconi M. Vibrational properties of crystalline Sb_2_Te_3_ from first principles. J. Phys. Condens. Matter 21, 095410 (2009). 21817396 10.1088/0953-8984/21/9/095410

[b37] Park H. *et al.* Consecutive and selective chemical vapor deposition of Pt/Al bilayer electrodes for TiO_2_ resistive switching memory. Jpn. J. Appl. Phys. 52, 10MC08 (2013).

[b38] Choi B. J. *et al.* Influence of substrates on the nucleation and growth behaviors of Ge_2_Sb_2_Te_5_ films by combined plasma-enhanced atomic layer and chemical vapor deposition. Chem. Mater. 21, 2386–2396 (2009).

[b39] Jeyachandran Y., Narayandass S., Mangalaraj D., Areva S. & Mielczarski J. Properties of titanium nitride films prepared by direct current magnetron sputtering. Mater. Sci. Eng. A 445–446, 223–236 (2007).

[b40] Simmonds M. G., Taupin I. & Gladfelter W. L. Selective area chemical vapor deposition of aluminum using dimethylethylamine alane. Chem. Mater. 6, 935–942 (1994).

[b41] Röder H., Hahn E., Brune H., Bucher J.-P. & Kern K. Building one- and two-dimensional nanostructures by diffusion-controlled aggregation at surfaces. Nature 366, 141–143 (1993).

[b42] Uccelli E., Nürnberger S., Bichler M. & Abstreiter G. & Fontcuberta i Morral, a. Growth mechanisms of self-assembled InAs quantum dots on (110) AlAs/GaAs cleaved facets. Superlattices Microstruct. 44, 425–430 (2008).

[b43] Madelung O. Semiconductors: Data Handbook. Polski tygodnik lekarski 14, (Springer Berlin Heidelberg, 2004).

